# Antioxidant and Anti-Fatigue Activities of Phenolic Extract from the Seed Coat of *Euryale ferox* Salisb. and Identification of Three Phenolic Compounds by LC-ESI-MS/MS

**DOI:** 10.3390/molecules180911003

**Published:** 2013-09-09

**Authors:** ChengYing Wu, Rong Chen, Xin Sheng Wang, Bei Shen, Wei Yue, Qinan Wu

**Affiliations:** 1 The School of Pharmacy, Nanjing University of Chinese Medicine, No. 138, Xianlin Avenue, Nanjing 210023, Jiangsu, China; E-Mails: wuchengying1979@gmail.com (C.Y.W.); kedingyu@126.com (R.C.); wxs501@gmail.com (X.S.W.); shen.bei.2008@163.com (B.S.); njyuewei@126.com (W.Y.); 2 Suzhou Institute for Drug Control, Suzhou 215104, Jiangsu, China; 3 Jiangsu Key Laboratory for TCM Formulae Research, Nanjing University of Chinese Medicine, No. 138, Xianlin Avenue, Nanjing 210023, Jiangsu, China

**Keywords:** *Euryale ferox* Salisb., phenolic extract, antioxidant activity, anti-fatigue activity

## Abstract

This study investigated the antioxidant potential and anti-fatigue effects of phenolics extracted from the seed coat of *Euryale ferox* Salisb. The *in vitro* antioxidant potentials, including scavenging DPPH, hydroxyl radical activities and reducing power were evaluated. Antioxidant status *in vivo* was analyzed by SOD, CAT, GSH-Px activities and the MDA content in liver and kidneys of D-galactose-induced aging mice. The anti-fatigue effect was evaluated using an exhaustive swimming test, along with the determination of LDH, BUN and HG content. The phenolic extract possessed notable antioxidant effects on DPPH, hydroxyl radical scavenging and reducing power. The mice which received the phenolic extract showed significant increases of SOD, CAT (except for in the kidney), GSH-Px activities, and a decrease of MDA content. The average exhaustive swimming time was obviously prolonged. Meanwhile, increase of LDH content and decrease of BUN content were observed after mice had been swimming for 15 min. The HG storage of mice was improved in the high and middle dose extract groups compared with the normal group. The contents of total phenols and gallic acid of the extract were determined. Three compounds in the extract were identified as 5,7-dihydroxy-2-(3,4,5-trihydroxyphenyl)-chroman-4-one, 5,7,4-trihydroxyflavanone and buddlenol E. These results suggest that the extract of *E. ferox* is a promising source of natural antioxidants and anti-fatigue material for use in functional foods and medicines.

## 1. Introduction

*Euryale ferox* Salisb., a large floating-leaf aquatic plant, is the only species in the genera *Euryale* of the family Nymphaeaceae and it is distributed in India, Korea, Japan, Southeast Asia and China, where it is mainly cultivated in Jiangsu, Shandong, Hunan, Hubei, and Anhui provinces [1]. It has been used as an important cash crop and as a valuable nourishing tonic in traditional medicine for centuries [2].

The seed of *E. ferox* has been applied in the treatment of diarrhea, spermatorrhea, and the petioles and pedicels in polydipsia, mouth dryness and dry throat [3]. The seed coat, which accounts for about half of the fruit weight, was usually discarded in large quantities after the seeds had been harvested. It represents one of the major waste products from the *E. ferox* production that nowadays has a scarce use or value. Previous studies reveal that the components of the seed coat mainly include tannins and polyphenols [4,5]. In preliminary experiments of our laboratory, optimal extraction conditions for the polyphenols in *E. ferox* seed coat has been investigated and the results show that the seed coat is comparatively abundant in polyphenols [5]. Polyphenols are widely distributed in the plant kingdom and have notable bioactivities, especially antioxidant activity [6,7,8]. In the human and animal diet, polyphenols from plant sources can protect against vascular lesions, cancers, diabetes [6,9,10,11] and have therapeutic effects in chronic fatigue [12]. These beneficial effects of polyphenols are mainly related to their antioxidant activity.

Recently, the search for natural antioxidants originated from plants instead of synthetic antioxidants has been a hot topic [6,10,11,13]. Natural antioxidants not only can be used for medicinal purposes, but also for food preservation, as dietary supplements or functional foods, and in cosmetics [14]. In this context, a variety of cheap waste products from the food or agricultural industries have been studied as potential sources of natural antioxidants for the environmental and economical benefits. Therefore, the *E. ferox* seed coat represents a potentially cheap source of natural antioxidants with a vast range of applications.

The literature data has demonstrated that the aqueous and alcoholic extract of *E. ferox* shells possess *in vitro* antioxidant activity [15], but the antioxidant potential of the seed coat has not been evaluated *in vivo*. Therefore, experiments for determining the possible use of *E. ferox* seed coat as natural antioxidant sources in an *in vivo* model are required. D-Galactose treatment causing oxidative stress has been used to induce aging model in mice for oxidative stress research [16]. Oxidative stress is a result of the imbalance between the antioxidant defense and the free radical production in the body, which can cause damage to cell membranes, protein, lipids and DNA and is involved in the development of aging, coronary heart disease and diabetes [17,18]. Researchers have also found that an important reason for physical or emotional fatigue is the increase in free radical formation thus causing oxidative damage to bodies [19,20]. The exhaustive swimming test has been used as an experimental exercise model to evaluate anti-fatigue compounds [21]. However, there is no report on the effects of the seed coat of *E. ferox* on physical fatigue. Some researchers have reported that the hydrolysis products of polyphenols extract in the seed coat was composed of gallic acid, chlorogenic acid, epicatechin, epicatechin gallate and rutin as analysed by HPLC-UV [22]. However, there are few studies on the identification of the phenolic compounds from the seed coat extract.

The main aims of this work were to evaluate the *in vitro* and *in vivo* antioxidant activity and *in vivo* anti-fatigue effect of the phenolic extracted from the seed coat of *E. ferox* with a view to its potential use in functional foods and medicines. In our study, the total phenolic content of the extract was determined by the Folin-Ciocalteu method, the content of gallic acid was determined by HPLC-UV/PDA, and the phenolic compounds in the extract were identified by HPLC-ESI-MS/MS. To the best of our knowledge, all this is reported for the first time.

## 2. Results and Discussion

### 2.1. *In Vitro* Antioxidant Activity Analysis

#### 2.1.1. DPPH Radical Scavenging Activity

**Figure 1:**
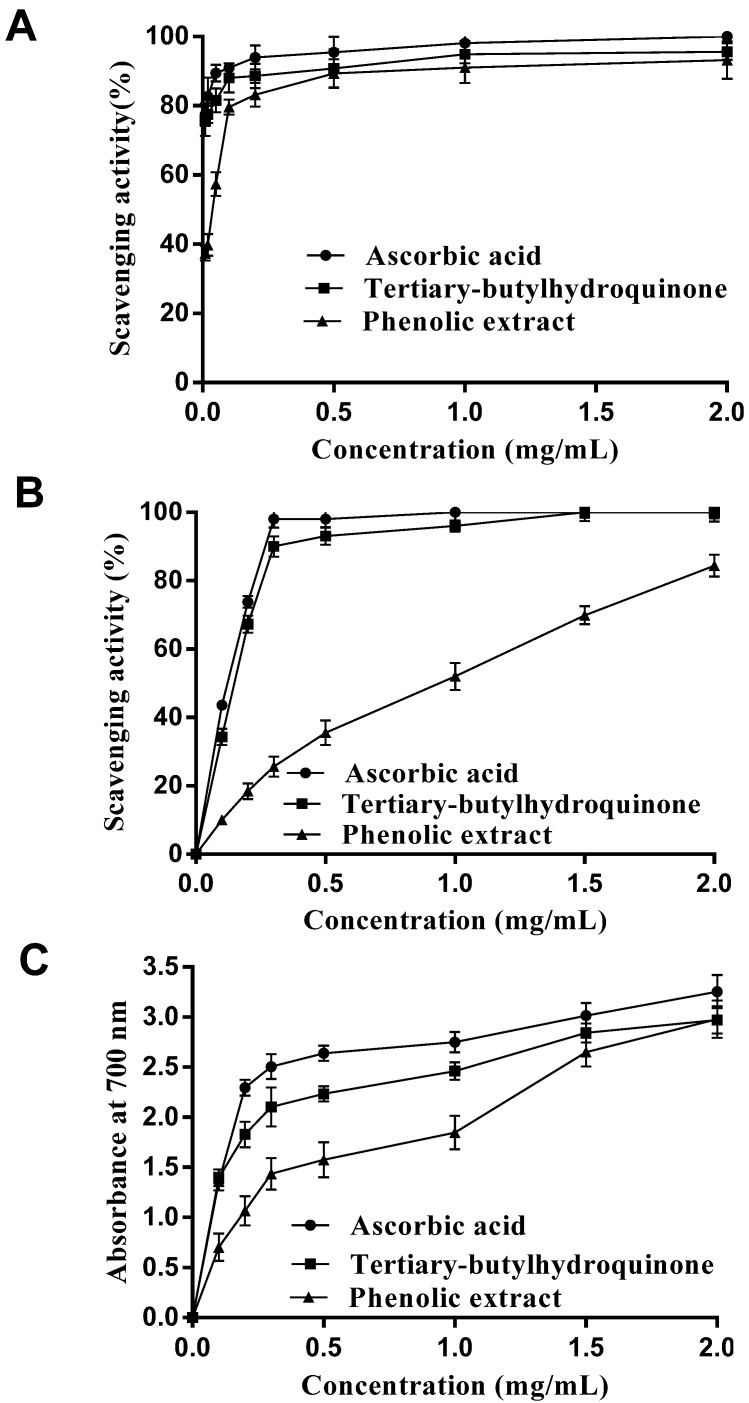
(**A**) DPPH radical scavenging activities, (**B**) hydroxyl radical scavenging activities and (**C**) reducing power of the phenolic extract and control standards. Results are expressed as a mean ± SD (n = 3).

The DPPH radical is a stable free radical at room temperature, which has been extensively used to evaluate the free radical-scavenging activity of antioxidants [23]. A lower absorbance of the reaction mixture indicates a higher DPPH radical-scavenging activity. The result of DPPH free radical-scavenging activity of the phenolic extract was shown in Figure 1A and compared with ascorbic acid and tert-butylhydroquinone as references. As shown in Figure 1A, the DPPH radical scavenging activity increased dramatically with the concentration from 0.01 to 0.5 mg/mL of the phenolic extract and was slightly lower than that of ascorbic acid and tert-butylhydroquinone at a concentration of 0.5 mg/mL. When the concentration of phenolic extract was >0.5 mg/mL, the DPPH radical scavenging activity increased slightly. The results indicated that the phenolic extract of *E. ferox* Salisb. seed coats had significant DPPH radical scavenging activity.

#### 2.1.2. Hydroxyl Radical Scavenging Activity

Hydroxyl radicals are strongly reactive oxygen species formed in biological systems that have been found to damage cellular components of DNA, proteins and lipids, resulting in many health problems such as cancer, aging, and cardiovascular disease [24]. In this study, hydroxyl radicals generated by the Fenton reagent were used to evaluate the scavenging activity of the phenolic extract. The results in Figure 1B show that the extract displayed antioxidant activity in a concentration-dependent manner, which suggested that the extract may contribute to prevent oxidative damage in the human body. The scavenging effect on hydroxyl radical, however, was weaker than that of ascorbic acid and tert-butyl-hydroquinone.

#### 2.1.3. Reducing Power

Reducing power is one of the mechanisms of antioxidant actions and may serve as a significant indicator of potential antioxidant activity [25]. The presence of the antioxidant results in the reduction of the Fe^3+^/ferricyanide complex to the Fe^2+^ form [26] and a chance of absorbance of the reaction mixtures. A higher absorbance indicates a stronger reducing power. As seen in Figure 1C, the phenolic extract displayed concentration-dependent reducing power, which was weaker than that of ascorbic acid and tert-butylhydroquinone.

### 2.2. *In Vivo* Antioxidant Activities Analysis

In this study, a D-galactose induced aging mice model was used for the evaluation of the *in vivo* antioxidant activity of the phenolic extract. The D-galactose induced aging animal model, which is established by consecutive subcutaneous D-galactose injections for approximately 6 weeks, has been frequently used in oxidative damage research [16]. Chronic injection of D-galactose can cause gradual deterioration in learning and memory capacity and activates oxidative stress in the brain of mice [27]. The primary endogenous antioxidant enzymes including SOD, CAT and GSH-Px, as well as a metabolic product of LPO (MDA) have been measured as the oxidative biomarkers for D-galactose-induced aging models [28]. These experiments showed that D-galactose significantly decreased the activities of antioxidant enzymes SOD, CAT and GSH-Px, and increased the MDA content in the liver and kidneys of mice compared with the normal animals. However, the phenolic extract exhibited the increase on the SOD, CAT, GSH-Px activities and decreasing content of MDA in the liver and kidneys, which indicated that this extract had efficient antioxidant property.

#### 2.2.1. Effect on the Activities of SOD

**Figure 2:**
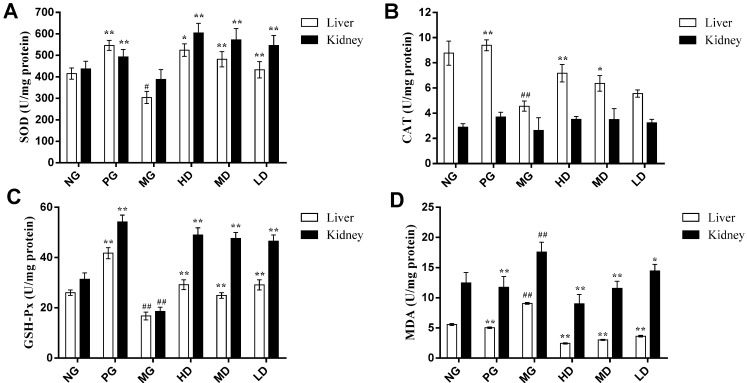
Effect of the phenolic extract on the SOD, CAT, GSH-Px activities and MDA content in the liver and kidney of aging mice induced by D-galactose. (**A**) SOD; (**B**) CAT; (**C**) GSH-Px; and (**D**) MDA. Results are expressed as a mean ± SD (n = 10). # *p* < 0.05, ## *p* < 0.01 compared with the normal group (NG). * *p* < 0.05, ** *p* < 0.01 compared with the model group (MG). PG: Positive control group; HD: High-dose extract treated group; MD: Middle-dose extract treated group; LD: Low-dose extract treated group.

SOD is a superoxide radical scavenging factor converted superoxide radicals to H_2_O_2_ [29]. Figure 2A shows the SOD activities in liver and kidney of mice after the experiment. Compared with normal group (NG), a decrease was observed for SOD activities of model group (MG). As shown in Figure 2A, SOD activities level of liver was significantly decreased on MG (*p* < 0.05) and that of kidney was not significant (*p* > 0.05) when compared with NG.

At the same time, the SOD activities in positive control group (PG) and three extract groups (respectively high-dose, middle-dose and low-dose extract groups) were significantly increased as compared with MG (*p* < 0.01 or *p* < 0.05). Moreover, the SOD activities of liver in three extract groups were lower than that of the PG and that of kidney were higher than that of the PG. However, Figure 2A indicates that the SOD activities were enhanced in three extract groups after treating with the phenolic extract. 

#### 2.2.2. Effect on the Activities of CAT

CAT catalyses the decomposition of H_2_O_2_ into H_2_O and O_2_ [30]. Figure 2B shows the CAT activities in liver and kidney of mice after the experiment. The CAT activities in liver of MG were significantly lower than NG (*p* < 0.01). In PG group, high-dose group and middle-dose group, CAT activities of liver were higher than MG (*p* < 0.01 or *p* < 0.05). In addition, a marked difference could not be found between low-dose and MG groups. Compared with NG, the CAT activities in kidney of MG were decreased without obvious variance. CAT activities of kidney in PG group and three extract groups were higher than MG group without obvious variance. Although, in D-galactose induced aging mice, administration of phenolic extract showed some efficacy in enhancement of CAT level but failed to show statistical significance.

#### 2.2.3. Effect on the Activities of GSH-Px

GSH-Px reduces H_2_O_2_ or hydroperoxides to H_2_O and alcohol [30]. Figure 2C represents the effect of phenolic extract on the GSH-Px activities in liver and kidney of aging mice. The GSH-Px activities of MG were decreased significantly compared with NG (*p* < 0.01). The treatment of aging mice with the phenolic extract and vitamin C led to increases in the GSH-Px activities. The PG and three extract groups showed significant increases in GSH-Px level compared with MG (*p* < 0.01).

#### 2.2.4. Effect on the Content of MDA

MDA is one product of LPO, which content reflects the damage to the cell membrane [31]. Figure 2D shows the levels of MDA in liver and kidney of aging mice. The MDA content of MG was increased very significantly more than NG (*p* < 0.01), both in liver and kidney. In MG, the MDA content of liver was very significantly higher than PG and the three extract groups (*p* < 0.01). Also, a significantly decreased level of MDA content in kidney was observed in PG and the three extract groups compared with MG (*p* < 0.01 or *p* < 0.05).

### 2.3. Anti-Fatigue Effect Analysis

Fatigue can be classified into mental and physical fatigue, which is involved in many physiological and biochemical factors [21]. One important reason for physical fatigue is the increase of free radicals. During physical fatigue status, the antioxidant defense system becomes weaker and is insufficient for completely preventing oxidative damage caused by excessive free radicals [19,20]. The exercise tolerance test is the most direct and objective indicator for reflecting physical fatigue [21]. In this study, an exhaustive swimming test was used to evaluate the exercise tolerance of mice, which was a direct measurement of an anti-fatigue effect [32,33]. Additionally, biochemical indicators related to fatigue including lactate dehydrogenase (LDH), blood urea nitrogen (BUN) and hepatic glycogen (HG) were determined after the mice had been swimming for 15 min. The results demonstrated that the phenolic extract prolonged the time to exhaustion during swimming, decreased the content of BUN and increased the content of HG, suggesting that the extract could alleviate fatigue in mice. This anti-fatigue effect might be related to the antioxidant properties of the sample. A possible explanation was that the sample was rich in phenolics, resulting in increased scavenging of free radicals, which prolonged the swimming time.

#### 2.3.1. Effect on Swimming Time to Exhaustion

**Figure 3:**
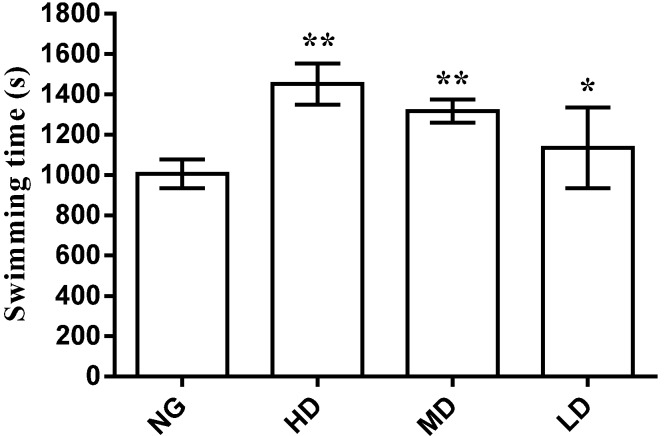
Effect of the phenolic extract on the swimming time in mice. Results are expressed as a mean ± SD (n = 10). * *p* < 0.05, ** *p* < 0.01 compared with the normal group (NG). HD: High-dose extract treated group; MD: Middle-dose extract treated group; LD: Low-dose extract treated group.

Anti-fatigue effects were correlated with longer swimming times. The results in Figure 3 show that the swimming time to exhaustion of the high and middle-dose extract groups were very significantly longer (*p* < 0.01) than for NG; the swimming time of the low-dose extract group was significantly prolonged (*p* < 0.05) compared with NG. These results indicated that the phenolic extract had significant effect on the endurance capacity of mice in this experiment.

#### 2.3.2. Effect on LDH and BUN Content

LDH and BUN are biochemical parameters related to fatigue. In relatively anaerobic exercise, lactic acid is accumulated more in the body, which results a decrease of the muscle strength and exercise-induced fatigue. LDH acts as a catalyst in the process of lactic acid clearance and it can effectively clear the lactic acid [34,35]. The results of the effect of phenolic extract on serum LDH shown in Table 1 indicated that no significant changes in the high and low-dose groups. However, the LDH content of middle-dose group increased but this was insignificant compared with NG (*p* < 0.05). There was a dose-independent effect in the three extract groups.

BUN is a product of energy metabolism, which is another indicator of fatigue status. Between BUN and exercise tolerance, there is a negative correlation. The less an animal is adapted to exercise, the more BUN increases [36]. As shown in Table 1, the BUN content in three extract groups was significantly lower than NG (*p* < 0.01). However, the effect was dose-independent in the three extract groups.

**Table 1:** Effects of *E. ferox* seed coat extract on LDH, BUN and HG levels in mice.

Group	*N*	Dose (mg/kg)	Serum		Liver
			LDH (U/L)	BUN (mmol/L)	HG (mg/g)
NG	10	−	3,583.01 ± 401.78	67.02 ± 4.66	3.39 ± 1.47
HD	10	400	3,397.33 ± 238.35	22.98 ± 3.30 **	6.61 ± 0.09 **
MD	10	200	3,638.64 ± 340.89	21.14 ± 5.69 **	7.51 ± 2.55 **
LD	10	100	3,257.25 ± 407.07	25.92 ± 5.79 **	2.67 ± 0.07 **

** *p* < 0.01, compared with normal group (NG).

#### 2.3.3. Effect on the Content of Hepatic Glycogen

Glycogen is an important source of energy during exercise. The stores of glycogen in the liver are related to the capacity for high intensive exercise. The increasing of glycogen stored in the liver can enhance the exercise endurance. Fatigue will happen when consuming hepatic glycogen. Therefore, liver glycogen is another index of fatigue [35,37]. As seen from Table 1, the hepatic glycogen content in the high and middle-dose extract groups was significantly higher compared with NG (*p* < 0.01). But in the low-dose extract group, it was lower than NG (*p* < 0.01). There is no obvious dose-dependence between the three extract groups. The result indicated that increased level of HG may be one of the pathways behind the phenolic extract’s anti-fatigue effect.

### 2.4. The Content of Total Phenolic and Gallic Acid

A larger number of experimental works have indicated that many polyphenols have *in vitro* and *in vivo* antioxidant activities and anti-fatigue effects. They can scavenge free radicals and increase the activities of antioxidant enzymes [6,7,8]. In this study, the total phenolic content of the phenolic extract was estimated using the Folin-Ciocalateu method. Under the extraction conditions described in Section 3.4, the yield of the crude extract was determined to be 113.30 mg GAE/g of dry weight. After purification by D101 macroporous resin adsorption, the phenolic extract was estimated to be 379.53 mg GAE/g of dry weight. The total phenolic content of the phenolic extract was increased by 234.87% more than in the crude extract.

**Figure 4:**
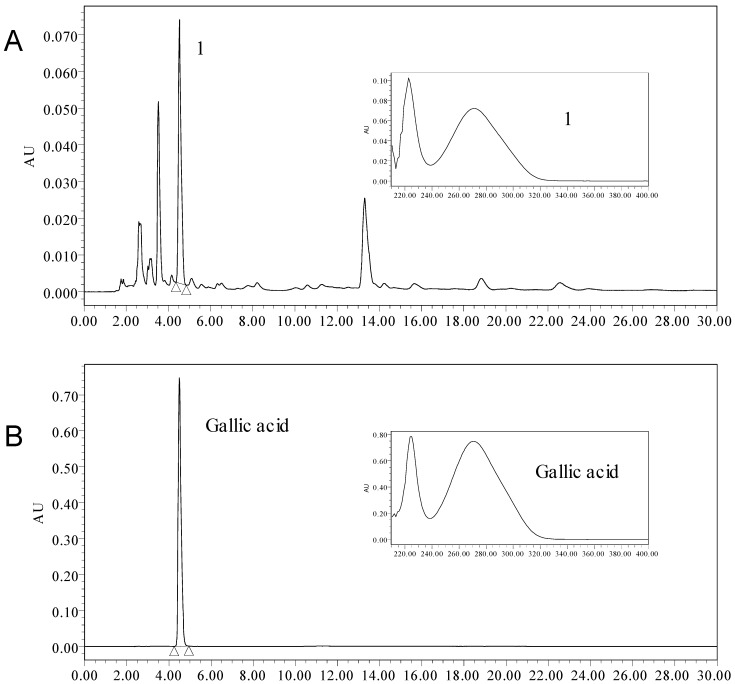
HPLC-UV/PDA chromatogram of the phenolic extract from seed coat of *Euryale ferox* Salisb.: (**A**) HPLC-UV/PDA chromatogram of the phenolic extract; (**B**) HPLC-UV/PDA chromatogram of Gallic acid.

Gallic acid is a plant polyphenolic compound widely found in many different plants. Studies have documented that gallic acid possess antioxidant properties [38,39,40,41]. In the present study, the content of gallic acid was detected using HPLC. By comparing the retention time and UV/PDA spectra of gallic acid with that in the phenolic extract, it can be concluded that gallic acid is a major component in the phenolic extract (Figure 4). The content of gallic acid was 138.15 mg per gram dry weight. This investigation demonstrated that the phenolic extract of *E. ferox* Salisb. seed coat was enriched in total phenolics and gallic acid content, which supports the efficient antioxidant and anti-fatigue properties of this extract.

### 2.5. Identification of Phenolic Compounds by LC-ESI-MS/MS

We carried out an analysis of the molecular ions (MS) and the main fragments of the different compounds obtained from the extract by LC-ESI-MS/MS. Three compounds were identified in the phenolic extract of *E. ferox* seed coat, which was in accordance with compounds already identified in the literature [42,43,44]. The compounds identified in the phenolic extract are listed in Table 2, which includes the retention times, molecular formulas and main fragments of each compound.

**Table 2:** Identification of the compounds in the extract by LC-ESI-MS/MS.

Compound Number	Retention time (min)	Molecular formula	[M−H]^−^ *m/z*	Main fragments *m/z*	Identification
1	11.813	C_15_H_12_O_7_	303.0510	151.0382	5,7-dihydroxy-2-(3,4,5-trihydroxyphenyl)chroman-4-one
2	14.386	C_15_H_12_O_5_	271.0584	151.0019 119.0495	5,7,4-trihydroxyflavanone
3	14.467	C_31_H_36_O_11_	583.2207	387.1420 357.1283 195.0636 165.0529	buddlenol E

**Figure 5:**
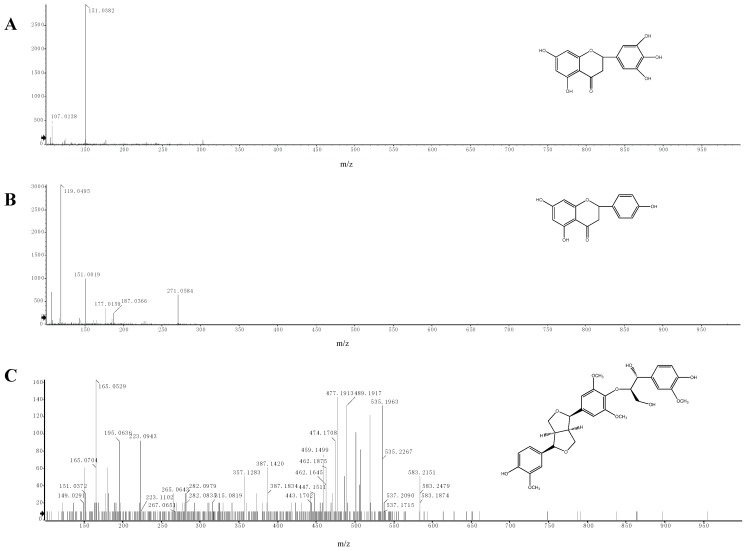
LC-ESI-MS/MS spectra of compounds in the phenolic extract. (**A**) 5,7-dihydroxy-2-(3,4,5-trihydroxyphenyl)chroman-4-one; (**B**) 5,7,4-trihydroxyflavanone; (**C**) buddlenol E.

In the negative mode, compound **1** showed the molecular ion [M-H]^−^ at *m/z* 303.0510 (C_15_H_11_O_7_) (Figure 5A). This compound fragmented according to retro-Diels Alder (RDA) reaction and the ion at *m/z* 151.0382 was the RDA fragment. Based on the fragmentation pattern and by comparison with known data in the seed of *E. ferox*, this compound was identified as 5,7-dihydroxy-2-(3,4,5-trihydroxyphenyl)chroman-4-one.

Compound **2** had a similar fragmentation pattern as compound **1**. It showed a molecular ion [M-H]^−^ at *m/z* 271.0584 (C_15_H_10_O_7_) in the negative mode (Figure 5B). The fragments at *m/z* 151.0019 and *m/z* 119.0495 corresponding to an RDA reaction were found. This compound was identified as 5,7,4-trihydroxyflavanone.

The [M-H]^−^ ion at *m/z* 583.2207 (C_31_H_35_O_11_) was assigned to buddlenol E (compound **3**, Figure 5C). According to the literature [34], the characteristic fragmentation of this compound produces ions at *m/z* 387.1420, 357.1283, 195.0636 and 165.0529.

## 3. Experimental Section

### 3.1. Plant Materials

Fresh fruit of *E. ferox* was collected from Suzhou City (Jiangsu Province, China) in September 2011, identified by the corresponding author, and deposited at the School of Pharmacy, Nanjing University of Chinese Medicine, Nanjing, China. The separated seed coat was shade dried and ground into powder for future use.

### 3.2. Chemicals and Reagents

Gallic acid was purchased from National Institutes for Food and Drug Control (Beijing, China); Folin-Ciocalteu reagent from Merck (Darmstadt, Germany). D-Galactose was purchased from the Beijing Chemical-Regent Company (Beijing, China). DPPH, ascorbic acid, tertiary-butylhydroquinone were purchased from Sigma-Aldrich (St. Louis, MO, USA). Assay kits (including Coomassie Brilliant Blue G-250, SOD, CAT, GSH-Px, MDA, LDH, BUN, and hepatic glycogen) were purchased from the Nanjing Jiancheng Bioengineering Institute (Nanjing, China). All other chemicals and reagents used in this study were of analytical grade and made in China. Experimental water was double distilled water.

### 3.3. Animals

Male Kunming mice (8 weeks old and weight of 18 to 22 g) were purchased from Shanghai Slac Laboratory Animals, Shanghai, China (animal license No. SCXK(HU) 2007-0005). All animals were housed at 25 ± 2 °C and 30%–60% relative humidity and were maintained on a 12-h light/12-h dark cycle. During the acclimatization period, mice were fed *ad libitum* with standard laboratory diet and water. All animal experiments conducted during this study had been approved by the ethics committee of Nanjing University of Chinese Medicine and in strict accordance with the Guiding Principles for the Care and Use of Laboratory Animals approved by the Animal Ethics Committee of China.

### 3.4. Extraction

Dried powder (50 g) of *E. ferox* seed coat was placed in a conical flask with 60% acetone solvent (1:50 w/v), and extracted two times using an ultrasonic bath working at a frequency of 40 kHz and the specified temperature of 25 °C for 30 min each time. Then the crude extract was filtered and purified using a column packed with D101 macroporous adsorption resin. Water and 10% (v/v) ethanol were used as desorption solvents. The eluate was collected and concentrated by rotary evaporation at 40 °C. Then, it was freeze-dried and kept at 4 °C for the analysis of total phenolic, antioxidant potential, anti-fatigue effect, and phenolic compounds identification by HPLC and LC-ESI-MS/MS.

### 3.5. *In Vitro* Antioxidant Activities

#### 3.5.1. DPPH Radical Scavenging Activity

The free radical scavenging activity of the phenolic extract was measured by DPPH test according to the method described by Zhang *et al.* [45] with some modifications. The sample was dissolved in water. Two milliliters of sample solution at various concentrations were mixed with DPPH (0.2 mM in 95% ethanol, 2 mL). The mixture was then shaken vigorously and kept at room temperature for 30 min in the dark. The absorbance was measured at 517 nm against a blank. Ascorbic acid and tert-butylhydroquinone at various concentrations were used as references. The experiment was performed in triplicate and averaged. The DPPH radical scavenging activity was calculated using the following equation:

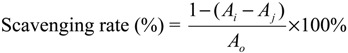
(1)
where *A_i_* is the absorbance of the sample with the DPPH; *A_j_* is the absorbance of the sample without DPPH; and *A_0_* is the absorbance of pure DPPH.

#### 3.5.2. Hydroxyl Radical Scavenging Activity

The hydroxyl radical scavenging activity of the phenolic extract was measured according to a literature method [35] with some modifications. A mixture of 1,10-phenanthroline (7.5 mM, 0.5 mL) and FeSO_4_ (7.5 mM, 0.5 mL) was mixed with sodium phosphate buffer (0.2 M, pH 7.4, 0.5 mL). Then, 1 mL of the sample at various concentrations and H_2_O_2_ (0.1%, 0.5 mL) were added. The mixture was incubated at 37 °C for 60 min, and the absorbance was measured at 510 nm. Ascorbic acid and tertiary-butylhydroquinone at various concentrations were used as references. The experiment was carried out in triplicate and averaged. The following equation was used:

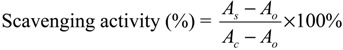
(2)
where *A_s_* is the absorbance of the sample; *A_0_* is the absorbance of the blank solution using distilled water; and *A_c_* is the absorbance of a control solution in the absence of H_2_O_2_.

#### 3.5.3. Reducing Power

The determination of reducing power was carried out as described by Wang *et al.* [46] with some modifications. Two milliliters of sample at various concentrations was mixed with phosphate buffer (0.2 M, pH 6.6, 2.5 mL) and potassium ferricyanide (1%, w/v, 2.5 mL). After the mixture was incubated at 50 °C for 20 min, trichloroacetic acid (10%, w/v, 2.5 mL) was added, and the mixture was centrifuged at 4,000 rpm for 10 min. The supernatant solution (5 mL) was mixed with ferric chloride (0.1%, w/v, 0.5 mL) for 10 min, and then the absorbance was measured at 700 nm against a blank. Ascorbic acid and tert-butylhydroquinone at various concentrations were used as references.

### 3.6. *In Vivo* Antioxidant Activities

#### 3.6.1. Experimental Design

After one week accommodation, the animals were randomly divided into six groups of 10 mice each. Aging mice model animals were induced by giving 0.2 mL of 400 mg/kg bw D-galactose dissolved in saline s.c. once daily for 42 consecutive days and the normal animals were injected equivalent saline only [47].

Group 1: Normal animals (normal mice treated with 0.5 mL of saline p.o. once daily from the 11th day to 42nd day);

Group 2: Model control animals (aging mice treated with 0.5 mL of saline p.o. once daily from the 11th day to 42nd day);

Group 3: Positive control animals (aging mice treated with 0.5 mL of 50 mg/kg vitamin C p.o. once daily from the 11th day to 42nd day);

Group 4: Low-dose extract treated animals (aging mice treated with 0.5 mL of 100 mg/kg bw phenolic extract dissolved in saline p.o. once daily from the 11th day to 42nd day);

Group 5: Middle-dose extract treated animals (aging mice treated with 0.5 mL of 200 mg/kg bw phenolic extract dissolved in saline p.o. once daily from the 11th day to 42nd day);

Group 6: High-dose extract treated animals (aging mice treated with 0.5 mL of 400 mg/kg bw phenolic extract dissolved in saline p.o. once daily from the 11th day to 42nd day).

#### 3.6.2. Biochemical Assay

At the end of the experiment, mice were sacrificed after an overnight fast. Liver and kidney of mice were collected and then floating blood was washed out with ice-cold saline, water was blotted with filter paper and finally the organs were weighed. Homogenates (10.0%, w/v) of liver and kidney were centrifuged at 5 °C, 3,000 r/min for 10 min in order to collect supernatant for further analysis. The protein level was determined by Coomassie Brilliant Blue method. The activities of SOD, CAT, GSH-Px and MDA content were assayed according to the recommended procedures provided by commercial reagent kits.

### 3.7. *In Vivo* Anti-Fatigue Effect

#### 3.7.1. Experimental Design

The animals were randomly divided into four groups based on body weight after one week accommodation, with 20 mice in each group. The normal group was given with 0.5 mL of saline p.o. once daily for 5 days. The extract groups included the high, the middle and the low dose group, which received 0.5 mL of 100, 200, and 400 mg/kg bw phenolic extract dissolved in saline p.o. once daily for 5 days, respectively.

Each group was further divided into two subgroups. One was used for the exhaustive swimming test; the other was used for collecting the blood and liver to determine biochemical parameters after swimming.

#### 3.7.2. Exhaustive Swimming Test

The exhaustive swimming test was carried out as described by Huang *et al.* [48] with some modifications. After the last treatment with extract or saline, forty mice were allowed to rest for 30 min. Then, they were placed in the swimming tank (30 cm × 30 cm × 50 cm) with 30 cm deep water at 25 ± 1 °C. A lead block (5% of body weight) was loaded on the tail root of the mice. The exhaustive swimming time was used as an index of the increase in exercise tolerance. The mice were determined to be exhausted when they failed to rise to the surface to breathe after 10 s.

#### 3.7.3. Biochemical Assay

After 24 h of the last treatment with extract or saline, forty mice were placed in the swimming tank (30 cm × 30 cm × 50 cm) with 30 cm deep of water at 25 ± 1 °C. After swimming for 15 min, they were taken out. Blood was collected from mice orbit to determine LDH and BUN content. The livers of the mice were taken to determine HG levels. All of the biochemical parameters were assayed according to the recommended procedures provided by commercial reagent kits.

### 3.8. Determination of Total Phenolic Content and Gallic Acid

The total phenolic content of the extract was determined according to the literature [49] using gallic acid solution as a reference to produce the calibration curve. The experiments were carried out in triplicate. The results were determined using the standard gallic acid calibration curve and expressed as mg of gallic acid equivalent (GAE) per gram of dry plant material.

A Waters 2695 chromatograph with a photodiode array detector and a C18 reversed-phase column (4.6 × 200 mm, 5 μm, Dalian Elite) was used for determination gallic acid of the extract [50]. The mobile phase consisted of methanol (solvent A) and 1% aqueous acetic acid solution (solvent B). Gradient condition was as follows: 0–6 min, 7% A; 6–8 min, 7%–14% A; 8–30 min, 14% A. Flow rate was 1.0 mL/min and injection volume was 10 μL. The temperature was fixed at 30 °C. Gallic acid was identified by comparing relative retention time and UV/PDA spectra with standard at 270 nm. Peak areas of the extract and standards were integrated from HPLC chromatograms by use of Waters Empowers software.

### 3.9. Identification of Phenolic Compounds by LC-ESI-MS/MS

The LC-ESI-MS/MS instrument consisted of a SIL-20A XR HPLC (a binary pump, a degasser and an autosampler) (Shimadzu, Kyoto, Japan) and a Triple TOF 5600 mass spectrometer (AB Sciex, Framingham, MA, USA), which was equipped with an electro spray ionization (ESI) source operating in the negative ion mode with spectra acquired over a mass range from *m/z* 10 to 1,000. ACQUITY UPLC HSS T3 column (2.1 × 100 mm, 1.8 μm, Waters, Milford, MA, USA) was used at flow rate of 0.3 mL/min. The injection volume was 5 μL. The column oven temperature was set at 40 °C. The mobile phase used was A: acetonitrile and B: 0.1% aqueous formic acid solution. Gradient program used was: 0–3 min, 5% A; 3–5 min, 5%–10% A; 5–10 min, 10%–20% A; 10–15 min, 20%–50% A; 15–20 min, 50%–90% A; 20–22 min, 90%–90% A; 22–23 min, 90%–5% A; 23–25 min, 5%–5% A. The ion spray source was 550 °C. The ion spray voltage was set at 5,000 V. The declustering potential was 80 V. Nitrogen was used as nebulizing and collision gas. The mass spectrometer was controlled by Analyst^®^ TF 1.6 software (AB Sciex) and the accurate mass data for the molecular ions were processed by PeakView^®^ 1.2.0.3 software (AB Sciex).

### 3.10. Statistical Analysis

Experimental results were processed using SPSS 18.0 (SPSS Inc., Chicago, IL, USA). The data were presented as mean ± standard deviation (SD) and analyzed using one-way ANOVA with Student’s *t*-test. Statistical significance was set at *p* < 0.05 or *p* < 0.01.

## 4. Conclusions

In our research, we used different *in vitro* and i*n vivo* antioxidant assays to examine the antioxidant activity and anti-fatigue effect of phenolic extract of *E. ferox* seed coat. According to the *in vitro* antioxidant assays, the phenolic extract exhibited scavenging effects on DPPH and hydroxyl radicals, and had notable reducing power. The antioxidant activities of the extract were further confirmed by carrying out *in vivo* assays. The reduction of SOD, CAT, GSH-Px activities and high level of MDA in liver and kidney of aging mice were observed compared with normal mice. Oral administration of the phenolic extract of *E. ferox* seed coat increased the activities of SOD, CAT, GSH-Px, and decreased the level of MDA in liver and kidney in aging mice compared with model control group. The anti-fatigue effect of phenolic extract from *E. ferox* seed coat was investigated. The phenolic extract prolonged the swimming time to exhaustion in mice. The phenolic extract treatment decreased the BUN content and increased the HG content of mice after swimming for 15 min compared with the model group. The LDH level was not affected by *E. ferox* seed coat extract in the experimental groups possibly because the experimental period was too short to promote such a modification. The extract of *E. ferox* seed coat was enriched in phenolic and gallic acid. 5,7-Dihydroxy-2-(3,4,5-trihydroxyphenyl)-chroman-4-one, 5,7,4-trihydroxyflavanone and buddlenol E. were identified in this extract. The antioxidant and anti-fatigue activities of this extract had a certain relationship with these compounds.

In conclusion, this study suggests that seed coat of *E. ferox* could be a potential and readily available source of natural antioxidants and might become a new functional food or medicine for fatigue resistance. In the future, we will continue the study on the chemical compounds and safety evaluation of the phenolic extract, in order to obtain valuable information for developing this product as an antioxidant and functional food.
